# Improving the information base regarding the health of people with a migration background. Project description and initial findings from IMIRA

**DOI:** 10.25646/5874

**Published:** 2019-03-14

**Authors:** Claudia Santos-Hövener, Maria Schumann, Patrick Schmich, Antje Gößwald, Alexander Rommel, Thomas Ziese, Thomas Lampert

**Affiliations:** Robert Koch Institute, Berlin Department of Epidemiology and Health Monitoring

**Keywords:** MIGRATION, MIGRATION BACKGROUND, HEALTH MONITORING, HEALTH REPORTING

## Abstract

Germany is an immigration country and nearly a quarter of its population has a migration background. Thus, there is increasingly a need for reliable information on the health situation of people with a migration background. The Robert Koch Institute is in charge of expanding its health monitoring to improve the representation of people with a migration background in interview and examination surveys. Studies adequately need to reflect the health status of people with a migration background and currently the Robert Koch Institute’s representative interview and examination surveys for adults do not fully achieve this. At the end of 2016, therefore, the Improving Health Monitoring in Migrant Populations (IMIRA) project was initiated aiming to expand the Robert Koch Institute’s health monitoring to people with migration background and improve their involvement in health surveys in the long-term. This includes carrying out two feasibility studies to test strategies to reach and recruit people with migration background for interview surveys and develop measures to overcome language barriers in examination surveys. In order to expand health reporting on migration and health, a reporting concept and a core indicator set will be developed and the potential of (secondary) data sources will be tested. Furthermore, plans foresee the testing and further development of relevant specific migration sensitive survey instruments and indicators, as well as increasing networking with relevant stakeholders.

## 1. Introduction

The Federal Republic of Germany is an immigration country. Nearly a quarter of the population has a migration background (23.6%), which means that either they themselves or at least one of their parents were born with a non-German citizenship [1]. Around half of the 19.3 million people with a migration background hold German citizenship (51.1%) and over two thirds (68.4%) have themselves migrated to Germany. The most common countries of birth of all people with a migration background in Germany are Turkey (2.8 million), Poland (2.1 million), the Russian Federation (1.4 million), Kazakhstan (1.2 million) and Romania (0.9 million) [1, 2]. In the group of migrants who do not hold German citizenship, the five most represented nationalities are Turkish, Polish, Syrian, Italian and Romanian [3].

People who migrate to Germany tend not to be sick more often, but their health resources and issues are different. These may vary greatly depending on region of origin and their experiences prior to, during and after migration. The heterogeneity of migrant groups with regard to factors such as culture and language, and also the causes of migration, as well as healthcare needs represent new challenges for the healthcare system [4, 5]. This makes it particularly important to gain information on the health status of current migrant groups and contribute to an analysis of health-related needs for integration [5, 6]. It will be equally important to improve the data basis on the health of people with a migration background who have already been in Germany for a longer time or were born here. Here too differences regarding protective factors are evident, for example lower levels of alcohol and tobacco consumption [7], and risk factors such as a less frequent utilization of health services [8]. Attempts to improve the data and information basis should therefore target the entire population with migration background. For the Robert Koch Institute the task will therefore consist of expanding the health monitoring the institute has developed over the past years, as well as expanding its health reporting which has become well established over the years to reach people with a migration background in national health surveys commensurate with their share of the German population and delivering representative statements on their health. The great challenge here lies in taking into account the diversity of people with a migration background, while at the same time ensuring data comparability.

**Info box**

**Health monitoring** at the Robert Koch Institute aims to continuously monitor disease incidence as well as health and risk behaviour in Germany. Moreover, the aim is to identify trends and changes in the health status and analyse these with regard to current or planned prevention measures. Health monitoring is conducted on behalf of the Federal Ministry of Health. Key elements of health monitoring at the Robert Koch Institute are the three health studies (1) German Health Interview and Examination Survey for Children and Adolescents (KiGGS), (2) German Health Interview and Examination Survey for Adults (DEGS1) and (3) German Health Update (GEDA). Starting in 2020, the next Germany-wide interview and examination survey for adults, the Health and Nutrition Survey in Germany (gern), will be conducted in co-operation with the Max Rubner-Institute.

**Federal health reporting** (GBE) regularly reports on the health of the German population. Federal health reporting provides a sound basis for political decision-making and offers a data-supported information base to all interested parties. It also serves to assess the success of measures and contributes to developing and evaluating health targets.

This article describes the Improving Health Monitoring in Migrant Populations (IMIRA) project that aims to expand health monitoring at the Robert Koch Institute to include people with a migration background and to improve their participation in health surveys in the long-term. The article also considers the measures and strategies applied in the context of previous German interview and examination surveys.

### 1.1 Including people with a migration background in health monitoring at the Robert Koch Institute so far

National-level health surveys of the adult population at the Robert Koch Institute have so far not satisfactorily taken people with a migration background into account. Regular surveys such as the German Health Update (GEDA) did not apply special measures to factor in this group. Based on this survey, there is therefore no robust data for people with a migration background. Furthermore, the German Health Interview and Examination Survey for Children and Adolescents (KiGGS) took considerable measures to include families with a migration background. With the aim of compensating for the low level of willingness to participate by families with a migration background, sampling in the KiGGS baseline study (2003-2006) was conducted by applying an oversampling factor of 1.5. Children and adolescents without German citizenship were therefore considered 1.5 as frequently in the unadjusted gross sample relative to their proportion of the population. Furthermore, using a computer-aided system of name categorisation (onomastic procedure) [9], participants with German citizenship were assigned to a particular language and therefore a possible migration background according to their names and surnames. People detected through onomastic sampling received translated invitation letters and survey materials in Turkish, Russian, Bosnian/Croatian/Serbian (Serbo-Croatian), Arabic, English and Vietnamese. Furthermore, field teams at the examination centres as well as the staff visiting the field prior to the survey received intercultural training. Specific public relations efforts for people with a migration background were implemented by using national and local level media published in these languages for example. Moreover, migrant organisations, integration commissioners (also for resettlers – ethnic Germans from Eastern Europe), counselling centres as well as the Working Group Migration and Public Health (Arbeitskreis Migration und öffentliche Gesundheit) at the Federal Commissioner for Migration, Refugees and Integration were informed in advance about the project and the initial findings [10]. The proportion of participants with a migration background in the unweighted sample was 17.0% (weighted 25.4%), although this proportion for 0- to 18-year-old children and adolescents with a migration background was lower than according to the 2005 Microcensus data (28.6%) [7, 11, 12].

**Figure 1:**
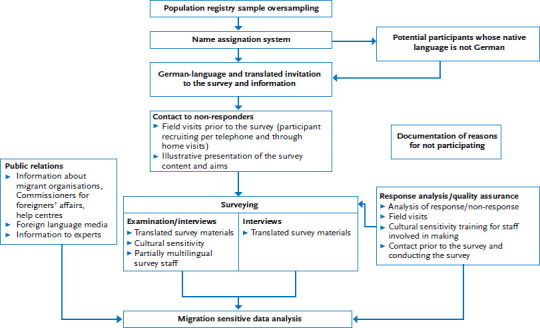
Improving the inclusion and participation of people with a migration background in KiGGS Wave 2 Source: Modified from Schenk et al. 2007 [15]

In the second follow-up to KiGGS (KiGGS Wave 2, 2014-2017), the migration specific approach of the KiGGS baseline study was further pursued and optimised (Figure 1). A total of 2,994 0- to 17-year-old children and adolescents with a migration background took part in KiGGS Wave 2. This corresponds to a proportion of 20.2% in the unweighted sample (weighted 28.8%), which was less than their proportion according to the 2013 Microcensus data (31.2%) [13].

In the context of the German Health Interview and Examination Survey for Adults (DEGS1) conducted between 2008 and 2011, some measures were implemented to include people with migration background. Thus, an oversampling by a factor of 1.5 of people with non-German citizenship, the translation of invitation letters and questionnaires into selected languages as well as specific public relations activities were implemented. However, due to limited resources, it was not possible to include all measures that proved successful in KiGGS. A total of 1,107 participants with a migration background were reached. This corresponds to an unweighted proportion of 14.2% of the net sample, which is below their share of 20.5% according to Microcensus data (2009). In DEGS1 specific subgroups with a migration background are underrepresented. Among them are migrants (first generation), women and men who hold Turkish citizenship, as well as people with low levels of education. A deeper analysis moreover shows distortions regarding length of stay and further sociodemographic and migration-specific variables in the context of DEGS1 [14].

Overall, it is evident that the response rates of children and adolescents and in particular adults of non-German nationality are lower compared to those of German nationals. This makes it particularly important to implement migration-specific measures to improve reach and participation and represent people with a migration background proportionate to their share of the population in the data. This is particularly true for hard-to-reach subpopulations, such as people with insufficient knowledge of German, first-generation immigrants, as well as people with low levels of education. Beyond translated invitation letters and survey materials, migration sensitive public relations efforts, visits to the field ahead of the survey have proven to be highly effective. Making personal contact and clarifying the aims and content of surveys appears to reduce barriers and fears and increase willingness to participate [10, 13, 15].

### 1.2 Heterogeneous data for describing the health status of people with a migration background

Beyond low response rates and possible barriers to participation, further challenges need to be addressed to improve the available data on the health of people with a migration background in the long term. More and more epidemiological studies provide data on the health of people with a migration background and numerous official statistics (Microcensus) and social science surveys such as the Socio-Economic Panel (SOEP) can be drawn upon for analyses to varying degrees. However, the different studies do not uniformly operationalise migration background, some rely on citizenship, others on country of birth, others consider parental country of birth. Comparing such data is therefore difficult. Moreover, it is often impossible to make statements on groups from specific regions or take into account variables such as length of stay, because the corresponding subsamples are too small. Simply differentiating by migration background (yes/no) however cannot do justice to the heterogeneity of people with a migration background. Ultimately, the situation calls for an established form of health reporting that regularly addresses the question of the health of people with a migration background and uses different data sources to gain a more complete picture.

## 2. The IMIRA project and initial results

In order to meet the challenges described above, in 2016 the Robert Koch Institute began the three-year Improving Health Monitoring in Migrant Populations (IMIRA) project that aims to expand health monitoring to also reach people with a migration background and increase their participation in health surveys in the long term. Results relevant to health monitoring are to be implemented during the next adult survey, the field phase of which is scheduled to begin in 2020. The survey can rely on the experiences and findings especially from the KiGGS studies. Beyond identifying relevant migration-specific concepts and indicators, an additional aim will be to expand health reporting. This will include a review and evaluation of the feasibility of using further sources of data, such as secondary data and SOEP data. Intensifying networking and co-operation with important national and international actors are also planned.

The IMIRA project consists of a total of eight subprojects (Table 1), which we describe below.

### 2.1 Taking stock of current research and adaptation of concepts (TP1 and TP2)

Subproject 1 took stock on the literature of ‘Migration and Health’ and provided an overview of current research at the national and international level. A review of the relevant literature identified publications dealing with ways of defining and operationalising migration background. Further studies that were included were those reflecting on forms of accessing and recruiting migrant populations through migration-specific surveying instruments and content. In addition, 26 experts such as researchers and stakeholders from the realms of academia and healthcare were interviewed to identify the challenges for epidemiological research in Germany as well as possible solutions for reaching people with a migration background. The results of these expert interviews showed several different challenges and strategies when approaching people with a migration background. Besides language and cultural barriers, a clear lack of trust in research exists, which can be met by including key migrant representatives and by using migrationsensitive translations [16].

Subproject 2 dealt with reviewing, developing and adapting the existing surveying instruments and concepts that can have an impact on the health of people with migration background. Within this context, the current operationalisation of a migration background in the adult surveys at the Robert Koch Institute was reviewed and harmonised. Additionally, the relevant literature regarding the application of the concept of acculturation in epidemiologic research was systematically reviewed. Acculturation describes a multi-dimensional process during which cultural practices, norms and values of the country of origin merge with those of the host country. Research has shown that this concept has an impact on the health of people with migration background [17-19]. Based on the results of the review, the aim is therefore to develop a short survey instrument for acculturation for the health surveys at the Robert Koch Institute. Further concepts relevant to migration were also identified and their collection and operationalisation discussed. Among them were subjectively perceived experiences with discrimination, surveying religious affiliation and subjective social status. In order to validate the concepts and tools people with a migration background were involved through focus groups and cognitive interviews.

### 2.2 Implementation of feasibility studies (TP3 and TP4)

Two feasibility studies were conducted in order to understand how to better reach people with a migration background in Robert Koch Institute surveys and therefore acquire comprehensive data on their health. The feasibility study entitled ‘interview survey’ (subproject 3) tested methodology with regard to their potential to increase the reachability of people with a migration background and increase this group’s response rates in health surveys. Data collection took place between January and May 2018. Using a population registry sample, people with a migration background in Berlin and Brandenburg were drawn for five groups based on the criterion of nationality (Croatian, Polish, Romanian, Syrian and Turkish) and subsequently received an invitation letter. In a sequential study design, different interview formats (online questionnaire, telephone interview and face-to-face interview) were offered to assess their acceptance. Prior to the study, the invitation letters and study information were developed with the help of people with migration backgrounds from the corresponding countries to verify the cultural sensitivity of all materials. Target persons received bilingual invitation letters and information brochures together with an invitation to participate in the study in the form of a bilingual online interview. Questionnaires were made available in German and participants’ mother tongues. For questions, or to cancel participation, target persons could call a multilingual study hotline. A first reminder letter offered the possibility of taking part in a telephone interview in addition to participating online. A final reminder letter sent to a random sample of the Turkish, Syrian and Romanian target persons notified home visits. During these visits, telephone numbers were collected to conduct a telephone interview, or a face-to-face interview was conducted on the spot. 1,090 participants filled out the questionnaires. The initial results show great differences in levels of willingness to participate, from 8.6% in the Turkish group to 24.3% in the Syrian group. All groups made use of the foreign language questionnaires, albeit with clear differences of preference. Around a quarter of people of Croatian nationality (23.6%), 41.0% of Turkish nationality, around half of the Polish people (51.1%) and Romanians (54.1%) as well as 80.9% of Syrian nationals used the non-German version. The feasibility study moreover showed that face-to-face interviews make it easier to reach people with low levels of education and at an older stage in life [20].

The feasibility study entitled ‘examination survey’ (subproject 4) aimed to test new methods for overcoming language barriers between the people being examined and those carrying out the examinations. The participation in examination surveys requires the written consent of participants, which is obtained after providing detailed information. So far, people without sufficient knowledge of German were excluded from examination surveys as it was not possible to provide them with adequate information on the content, counter indications of examinations and questions concerning data protection, due to their lack of or insufficient knowledge of German.

From October to December 2017, different measures to overcome language barriers were tested and evaluated for their application in the forthcoming adult survey in 2020. Around 90 participants respectively with a Polish, Syrian and Turkish migration background were recruited in a non-random sampling procedure (convenience sampling). Participants with low levels of German, based on the European Council reference framework for languages (B1 or less), were explicitly selected for the examination survey. The feasibility study here relied on information videos in German and the person’s mother tongue as well as multilingual information materials. A video interpreting service was available to provide information on the content of the study prior to participants providing their written consent and for any additional questions during the examination. Information letters and questionnaires were offered in German, Arabic, Polish and Turkish. A final evaluation of the measures applied by participants was also available whenever this was necessary with a video interpreting service. Following the feasibility study ‘examination survey’, focus groups in the corresponding languages were conducted with selected participants, to speak about the use of methods and overcoming barriers during the participation in surveys. The initial results indicate a high level of acceptance in particular of the multilingual information videos and video interpreting service. The examination and interview survey for adults due to begin in 2020 will therefore use translations, a video interpreting service as well as information videos in selected languages. In future, this will allow also people with low levels of German to participate in Robert Koch Institute health surveys. Due to the positive experiences made in the context of the IMIRA project, videos will also be used for the German language population and therefore provide a standardised tool of information provision.

### 2.3 Expanding health reporting (TP5 – TP7)

Federal health reporting regularly reports on the health of the German population. Health monitoring data here provide an important basis. Many further sources of data are also used to provide a report as complete as possible on the questions at hand. Within the context of the IMIRA project, a reporting concept is being developed that supports regular reporting on the health of people with a migration background (subproject 5). One question being analysed is, for example, the format future reports on the health of people with a migration background should have (standalone health report versus migration as a cross-sectional issue within broader reporting formats). To develop a relevant reporting concept for migration, international best practice examples are being identified. Beyond systematic research online, an online survey targeting national public health institutes and further relevant institutions in EU and OECD countries was conducted. Within the context of the subproject, a set of core indicators for describing the health of people with a migration background is being developed. Further data beyond health monitoring that could be used in an expanded health reporting is being reviewed. In addition to defining thematic focuses, stock has been taken of the national surveys in Germany that adequately collect information relating to health and migration background.

Within the context of the project, the question, as to what extent secondary data (e.g. data from statutory health insurances) can be used and the explanatory power and reliability such data has regarding the health of people with migration background (subproject 6). Secondary data is not collected primarily for scientific, rather for administrative purposes, but is, however, frequently used for scientific purposes. To get an overview, stock was broadly taken of the available data sources and the options for analysis they offer. In a second step, two sources of data that were considered as particularly relevant were looked at more closely and evaluated (asylum-seeker benefits statistics and cause-of-death statistics). Furthermore, data from the Socio-Economic Panel (SOEP) is being used for an evaluation project (subproject 7). SOEP is a representative longitudinal study to collect data on political and social change in Germany. While health is not a focus within SOEP, health-related questions have increasingly become part of the surveying programme over the course of the last five years. Within the context of SOEP, different populations with migration background have explicitly been taken into account since 1984. In 2016, for example a new and comprehensive sample of refugees in Germany was drawn up and initial interviews conducted [21].

### 2.4 Networking with relevant stakeholders (TP8)

A further subproject across the entire duration of the project has aimed to network and create co-operation with important stakeholders in Germany and Europe (subproject 8). The aim is to network with university institutes and public health practice. This includes a joint presence at congresses and conferences, scientific supervision of other migrant and/or refugee and health-related projects, as well as involving public health services and migrant organisations. An advisory board accompanies the project and provides guidance.

## 3. Conclusion

The IMIRA project followed the aim of establishing migration-sensitive health monitoring at the Robert Koch Institute that does justice to the heterogeneity of the population with migration background in Germany. This is essential for providing reliable data on the health situation and behaviour of people with migration background in the future. To provide up-to-date and robust data in the context of health monitoring that provide meaningful information on people with migration backgrounds in its full diversity, it is crucial to representatively include these populations. In future, the aim is to be able not only to account for migration background or generation, but also to reflect aspects such length of stay, residency status, level of German or region of origin and ensure a differentiated analysis of the health situation of people with migration background.

Based on the experiences made in previous interview and examination surveys and the initial results from the IMIRA project, the following elements are essential for reaching and representing people with migration backgrounds within the context of health monitoring:

**►** further developing the survey content, indicators and concepts to do justice to the heterogeneity of people with migration backgrounds
**►** offering greater flexibility in the choice of different interview formats
**►** ensuring personal contact in data collection, in particular face-to-face interviews to include groups which are particularly hard-to-reach
**►** focusing on overcoming language barriers by using multilingual services, materials and offering (video) interpretation services in examinations
**►** taking into account concepts such as ‘diversity’ and intercultural competency and therefore the use of migration-sensitive materials, as well as offering training sessions for the survey and research staff
**►** involving people with migration backgrounds in the planning, implementation and dissemination of results and promoting participation
**►** further developing health reporting and a reflected presentation of migration-specific statements

The coming national interview and examination survey for adults, the Health and Nutrition Survey in Germany (gern), that the Robert Koch Institute will conduct in cooperation with the Max Rubner Institute, will implement the conclusions from IMIRA regarding improving the reachability of people with migration background. The aims will therefore be (1) to reach adults with migration background commensurate with their share of the population; (2) to enable differentiated statements on the health situation of different migrant groups. The gern study applies established measures to increase response (visiting the field prior to the survey and public relations). Language barriers during examinations are to be addressed by using multilingual materials and information videos and making video interpretation services available. To provide findings on the health status of specific groups depending on their region of origin, the aim is to achieve a sample of non-German nationals for interviews from five select countries of origin (1,000 to 1,500 people per group). Interviews will be based on multilingual online and paper questionnaires, as well as face-to-face interviews.

## Key statements

- Interview and examination surveys of the Robert Koch Institute so far do not represent people with a migration background proportional to their share of the population.
- The IMIRA project was initiated to include people with a migration background in health monitoring at the Robert Koch Institute in the long term.
- Overcoming language barriers, applying different interview formats and culturally sensitive invitation letters are key to reaching the target group.
- Consulting with people with a migration background improves the quality of materials such as invitation letters or survey information.
- Analyses of the health status of people with a migration background should cover several aspects such as length of stay, residency status, German language competency or region of origin.

## Figures and Tables

**Table 1:** Subprojects of the IMIRA project Own table

Subproject	Inhalte
TP1	Taking stock of current research
TP2	Adaptation and further development of concepts
TP3	Feasibility study ‘interview survey’
TP4	Feasibility study ‘examination survey’
TP5	Further development of health reporting
TP6	Utilization of secondary data
TP7	Utilization of SOEP data
TP8	Networking and co-operation

TP = subproject, SOEP = Socio-Economic Panel
